# Metabolism of aromatics by *Trichosporon oleaginosus* while remaining oleaginous

**DOI:** 10.1186/s12934-017-0820-8

**Published:** 2017-11-17

**Authors:** Allison Yaguchi, Alana Robinson, Erin Mihealsick, Mark Blenner

**Affiliations:** Department of Chemical and Biomolecular Engineering, Clemson University, 206 S. Palmetto Blvd., Clemson, SC 29634 USA

**Keywords:** *Trichosporon oleaginosus*, Aromatics, Phenol, Resorcinol, p-Hydroxybenzoic acid, Lignin, Yeast, Aromatic metabolism

## Abstract

**Background:**

The oleaginous yeast, *Trichosporon oleaginosus*, has been extensively studied for its ability to metabolize non-conventional feedstocks. These include phenol-containing waste streams, such as distillery wastewater, or streams consisting of non-conventional sugars, such as hydrolyzed biomass and various bagasse. An initial BLAST search suggests this yeast has putative aromatic metabolizing genes. Given the desirability to valorize underutilized feedstocks such as lignin, we investigated the ability of *T. oleaginosus* to tolerate and metabolize lignin-derived aromatic compounds.

**Results:**

*Trichosporon oleaginosus* can tolerate and metabolize model lignin monoaromatics and associated intermediates within funneling pathways. Growth rates and biomass yield were similar to glucose when grown in 4-hydroxybenzoic acid (pHBA) and resorcinol, but had an increased lag phase when grown in phenol. Oleaginous behavior was observed using resorcinol as a sole carbon source. Fed-batch feeding resulted in lipid accumulation of 69.5% on a dry weight basis.

**Conclusions:**

Though the exact pathway of aromatic metabolism remains to be determined for *T. oleaginosus*, the results presented in this work motivate use of this organism for lignin valorization and phenolic wastewater bioremediation. *Trichosporon oleaginosus* is the first yeast shown to be oleaginous while growing on aromatic substrates, and shows great promise as a model industrial microbe for biochemical and biofuel production from depolymerized lignin.

**Electronic supplementary material:**

The online version of this article (10.1186/s12934-017-0820-8) contains supplementary material, which is available to authorized users.

## Background

Valorization of lignocellulosic biomass wastes is critical for the economic viability of the biomass economy [1]. Furthermore, valorization of wastewater and food and agricultural wastes may enhance sustainability and provide additional economic benefits [1, 2]. Waste streams are often heterogeneous in nature and contain additives and by-products, such as phenolics, that are toxic to human health. A by-product or waste that has gained considerable attention recently is lignin. It is the second-most abundant biopolymer on Earth and the only renewable, readily-available biopolymer comprised of aromatics [1]. Lignin is a by-product of biomass used as a feedstock for biofuels and biochemical production, and is a prominent by-product of pulp and paper mills. Kraft lignin is typically burned for its heating value, and only 2% is recovered for nonfuel uses [3]. Rather than burning it, lignin could be utilized in biorefineries as a feedstock for microbial production of higher value products [2, 4]. In addition, the large quantities of aromatics in industrial wastewater effluents makes aromatic compounds a prime target for waste valorization [5].

Bacterial metabolism and growth on various phenolic compounds is well-characterized. Many of these aromatics are representative lignin hydrolysate compounds or common products of lignin depolymerization [6]. Significant work has characterized phenolic metabolism in *Rhodococcus opacus* PD630 [7–9], *Acinetobacter baylyi* ADP1 [10, 11], and *Pseudomonas putida* [4, 12–15]. There are fewer examples of yeast that have been characterized to grow on phenolics, such as *Pichia holstii* [16], *Candida tropicalis* [17], and *Trichosporon cutaneum* [18]. These yeasts have evolved to handle a wide variety of aromatic substrates, utilizing so-called funneling pathways to convert diverse molecular species into a small number of metabolites [19]. There are many pathways for aerobic aromatic metabolism, with the ortho- and meta-cleavage pathways being most common [19]. These pathways generate central metabolites, such as pyruvate and acetyl-CoA [20–23]. Understanding the biochemical pathways of aromatic metabolizing organisms enables downstream engineering for high value products, such as oleochemicals for pharmaceutical, fuel, and specialty chemical applications.

Oleaginous microorganisms are a rational starting point for microbial production of oleochemicals, such as lipids for biofuels and omega-3 fatty acids. These microbes are characterized by their capacity to accumulate at least 20% of their mass as lipids. Significant attention has been given to several oleaginous yeast, including *Yarrowia lipolytica* [24–27], *Lipomyces starkeyi* [28], and *Rhodosporidium toruloides* [29]. Substantial work has been done to expand the genetic engineering tools available for these non-conventional oleaginous yeasts, enabling metabolic engineering of these species [30–36]. Despite being established industrial hosts with significant prior work, these yeast species are not suitable for utilizing certain low-cost feedstocks, such as aromatic-rich lignin and phenolic wastewater streams. Given the 50 million tons of lignin currently produced per year [3], there is a great need for microorganisms that are able to tolerate and even metabolize aromatic feedstocks. Of the oleaginous microorganisms that tolerate aromatic toxicity, bacteria do not achieve a high enough biomass and are prone to phage infection, and white-rot fungi grow too slowly. Similarly, few oleaginous yeasts are known to metabolize aromatics. Furthermore, the oleaginous yeast shown to metabolize aromatics do not maintain high lipid accumulation under aromatic growth conditions.

This study addresses the narrow crossover between efficient aromatic metabolism, rapid growth kinetics, and high endogenous lipid accumulation by investigating *Trichosporon oleaginosus,* a non-model, non-conventional yeast previously known as *Cryptococcus curvatus*. A recent review summarizes the ability of *T. oleaginosus* to metabolize a number of non-conventional feedstocks and maintain oleagincity [37]. In this study, we found *T. oleaginosus* tolerates several aromatics and metabolizes them when used as a sole carbon source. Simultaneous growth on mixtures of sugars and aromatics appeared diauxic; however, both substrates were completely consumed. Finally, we demonstrate fed-batch growth on aromatics results in over 69% of biomass accumulated as lipids in a simple shake flask. Due to its rapid growth rate on aromatics, and its significant lipid accumulation, we suggest *T. oleaginosus* has great potential as a model system for aromatic metabolizing oleaginous yeast.

## Results

### *Trichosporon oleaginosus* tolerates model lignin-derived aromatics

**Fig. 1:**
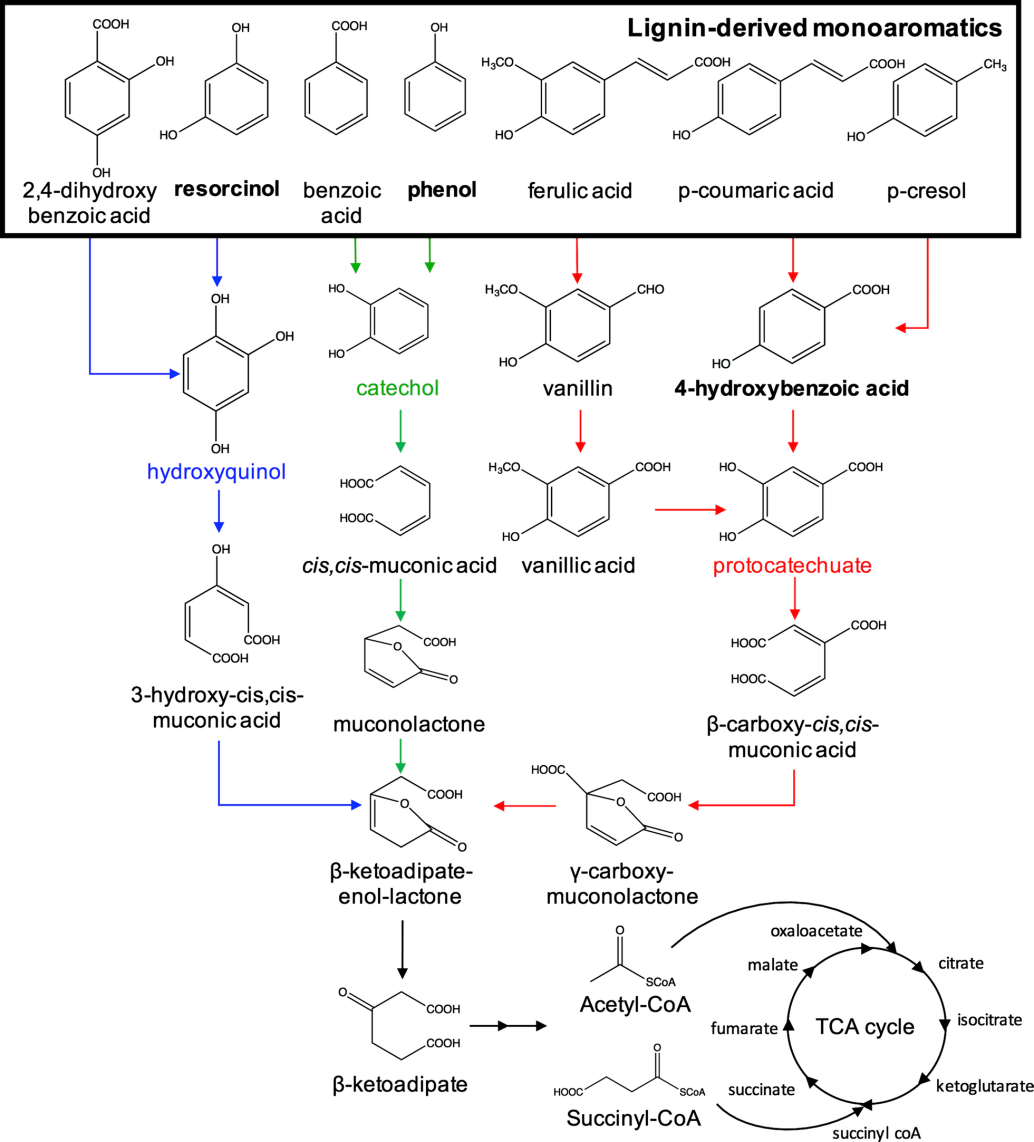
Funneling pathways for ortho-cleavage aromatic metabolism. The bolded box contains monoaromatic compounds often found in lignocellulosic hydrolysates. The three main funneling pathways for aromatic metabolism are the hydroxyquinol (blue), catechol (green), and phenol (red) pathways. *Trichosporon oleaginosus* can tolerate and metabolize many of the intermediates associated with the funneling pathways of aromatic compounds, and representative compounds were chosen for further characterization. Aromatic substrates of main focus in this work (resorcinol, phenol, 4-hydroxybenzoic acid) are bolded for emphasis

A BLAST search of aromatic metabolism genes against the *T. oleaginosus* genome returns several putative ortho-cleavage enzymes and no hits from the meta-cleavage pathway, suggesting aromatic metabolism may use the ortho-cleavage route (Fig. 1) [4]. Given its potential for aromatic metabolism, and its known tolerance to several inhibitors, we reasoned that *T. oleaginosus* was likely to tolerate many aromatic compounds. Therefore, 15 model aromatic compounds were screened to determine the minimum inhibitory concentration (MIC), which is the minimum concentration completely inhibiting cell growth (Table 1). Notably, protocatechuate had the highest MIC at 15.5 g/L for *T. oleaginosus*. The next highest MIC value was for hydroxyquinol at 7.5 g/L, followed by resorcinol at 5.5 g/L. Other compounds with MIC values over 1 g/L included: 2,4-dihydroxybenzoic acid (1.5 g/L), catechol (3.0 g/L), *p*-coumarate (1.4 g/L), *p*-hydroxybenzoic acid (4.0 g/L), and phenol (1.2 g/L).

**Table 1:** Minimum inhibitory concentrations (MICs) for compounds commonly found in lignin hydrolysates and funneling pathways for aromatic metabolism

Compound	MIC (g/L)
2,4-dihydroxybenzoic acid	1.5
Benzoic acid	1.4
Catechol	3.0
Ferulic acid	0.6
Guaiacol	1.5
Hydroxyquinol	7.5
*p*-Coumaric acid	1.4
*p*-Cresol	0.8
pHBA	4.0
Phenol	1.2
Protocatechuate	15.5
Resorcinol	5.5
Syringic acid	2.0
Vanillic acid	1.6
Vanillin	0.2

### *Trichosporon oleaginosus* metabolizes model lignin-derived aromatics

**Fig. 2:**
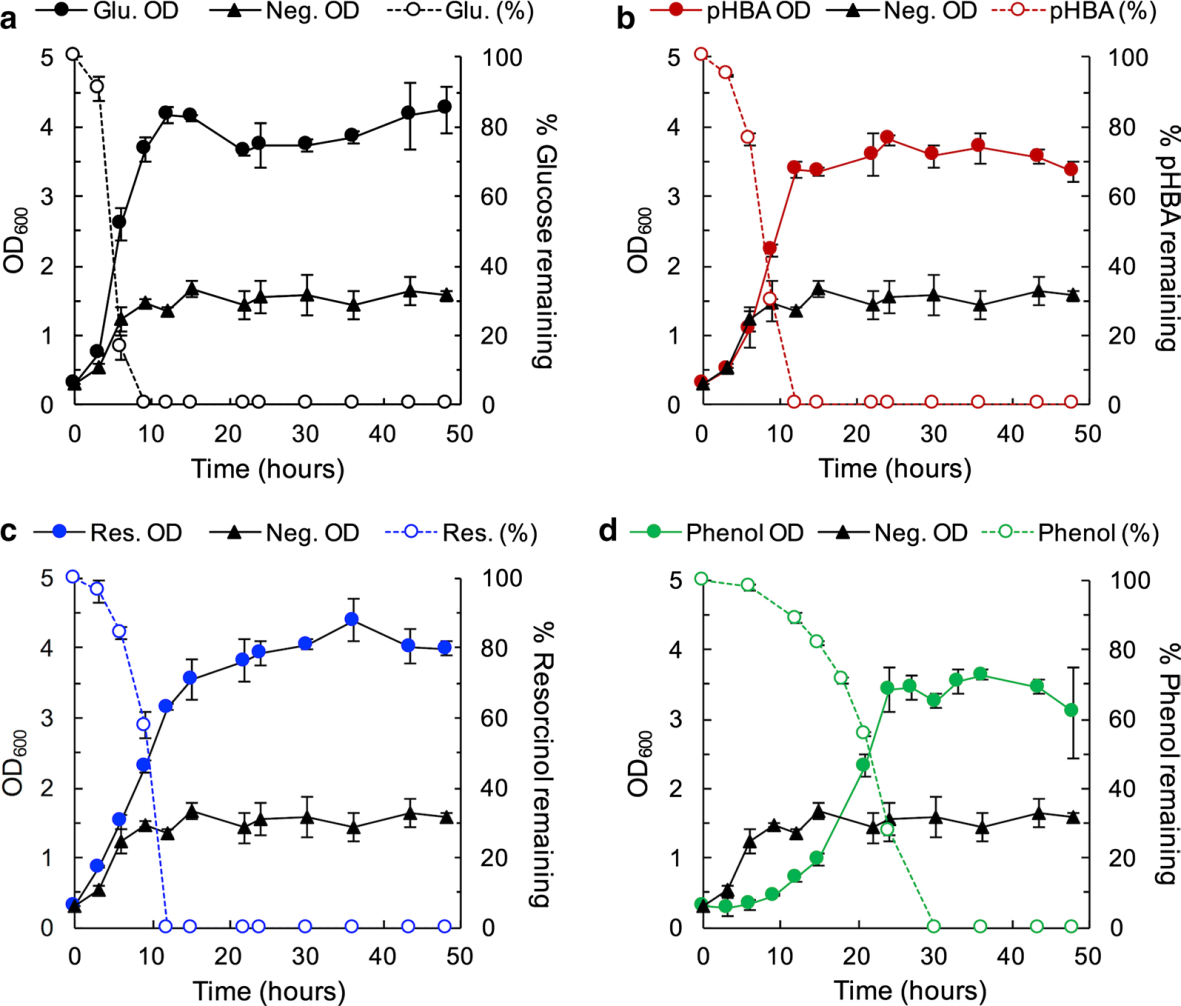
*Trichosporon oleaginosus* can metabolize aromatic substrates. Growth (closed circles or triangle, solid lines) and substrate utilization (open circles, dashed lines) data for 1 g/L **a** glucose, **b** pHBA, **c** resorcinol, and **d** phenol in TOHN media, respectively. The left axis represents OD_600_ while the right axis represents percent substrate remaining in the media. Growth in 1 g/L of carbon is compared to a negative control containing no additional carbon source (closed triangle, solid line). Each panel shows full consumption of the aromatic compound. Cells grown in resorcinol and pHBA have growth rates approaching those grown in glucose, whereas cells grown in phenol had a longer lag phase and a slower growth rate. Substrates were chosen as representative compounds for each funneling pathway shown in Fig. 1. The data in **a**–**d** are the mean and error bars are standard deviation of biological replicates (n = 3). Lines are used only for visual aid

The tolerance to a number of different compounds suggests *T. oleaginosus* may be a good candidate for aromatic metabolism. To determine if this yeast has a natural metabolism of aromatic compounds, we chose three aromatics for further analysis—phenol, resorcinol, and 4-hydroxybenozic acid (pHBA). Resorcinol was picked over 2,4-dihydroxybenzoic acid because it had a higher MIC. Phenol was picked over benzoic acid because of the broad interest in phenol-containing waste stream in industry and similar MIC. pHBA was picked over all other compounds upstream of protocatechuate because it had the highest MIC. Each aromatic serves as a representative compound for a branch of the funneling pathways shown in Fig. 1. As a stringent test of metabolism, we picked compounds upstream in the pathway, but with tolerance high enough to allow for significant lipid accumulation. *Trichosporon oleaginosus* cells were grown in a minimal high nitrogen (TOHN) and low nitrogen (TOLN) media with 1 g/L added carbon source (phenol, resorcinol, pHBA, or glucose). Growth curves shown in Fig. 2 represent growth in TOHN and are compared to a no-carbon-added negative control, which accounts for growth attributed to the small amount of yeast extract in the media. Media containing added carbon, whether glucose or aromatics, shows growth greater than the negative control, demonstrating that these substrate are metabolized for growth. All cells grown in media with an added carbon source reach similar biomass titers, indicating that aromatic compounds do not deleteriously affect total biomass accumulation. Cells inoculated into resorcinol and pHBA-containing media grow similarly to each other and somewhat slower to cells inoculated into glucose, with specific growth rates for glucose, resorcinol, and pHBA of 0.157 ± 0.016, 0.0965 ± 0.012, and 0.092 ± 0.001 h^−1^, respectively. Phenol induces a slower specific growth rate of 0.058 ± 0.002 h^−1^ and has a longer lag phase, as cells did not enter exponential phase until 12 h after inoculation. The negative control has a growth rate of 0.101 ± 0.010 h^−1^. Substrate utilization data, as measured by HPLC–UV-vis, indicate that all the compounds were fully consumed by 9 h for glucose, 12 h for resorcinol and pHBA, and 30 h for phenol. In the high nitrogen media, lipid accumulation was slightly lower in phenol (5.26 ± 0.09% w/w) as compared to resorcinol (7.21 ± 0.21% w/w), pHBA (7.55 ± 0.24% w/w), and glucose (6.72 ± 0.29% w/w) (Table 2). In low nitrogen media, lipid accumulation was slightly lower; however, given the toxicity limit for aromatic substrates, we were unable to create nitrogen limited condition needed for lipid accumulation using high concentrations of carbon in growth experiments. Furthermore, a small amount of yeast extract was needed for *T. oleaginosus* growth, limiting the achievable carbon to nitrogen (C:N) ratio to 12:1, assuming 11.1% nitrogen in the BD technical bacto yeast extract (reported in the BD bionutrients technical manual).

**Table 2:** Measurement of dry cell weight, lipid titer, and percentage lipid accumulation in high nitrogen (TOHN) and low nitrogen (TOLN) media containing 1 g/L carbon

Substrate	N conc. (g/L)	Dry cell weight (g/L)	Lipid titer (g/L)	Lipid accumulation (%)
Glucose	4	0.71 ± 0.00	0.05 ± 0.00	6.72 ± 0.29
Resorcinol	4	0.70 ± 0.06	0.05 ± 0.00	7.21 ± 0.21
pHBA	4	0.64 ± 0.05	0.05 ± 0.00	7.55 ± 0.24
Phenol	4	0.80 ± 0.01	0.04 ± 0.00	5.26 ± 0.09
Negative	4	0.22 ± 0.03	0.03 ± 0.00	12.22 ± 1.20
Glucose	0.012	0.96 ± 0.02	0.05 ± 0.01	5.44 ± 1.04
Resorcinol	0.012	0.66 ± 0.01	0.03 ± 0.01	4.67 ± 1.24
pHBA	0.012	0.69 ± 0.02	0.03 ± 0.00	4.72 ± 0.23
Phenol	0.012	0.80 ± 0.01	0.05 ± 0.00	6.13 ± 0.25
Negative	0.012	0.28 ± 0.00	0.02 ± 0.00	6.11 ± 0.69

Errors are reported as standard deviation of biological triplicates

GC-FID was used to determine the fatty acid composition of cellular lipids (Table 3). This study agrees with previous reports that this yeast accumulates high amounts of linoleic acid and α-linolenic acid when grown in glucose [38, 39]. However, cells grown with resorcinol, pHBA, and phenol show similar amounts of linoleic acid, an increase in oleic acid, and decrease in α-linolenic acid (Table 3). When cells are grown in low nitrogen glucose-containing media, lipid accumulation profiles show an increase in palmitic, palmitoleic, stearic, and oleic acid and a decrease in linoleic and α-linoleic acid. In low nitrogen aromatic containing media, the lipid profiles are change in a substrate-dependent manner. For pHBA, low nitrogen resulted in increased palmitic acid, with small decreases to palmitoleic, stearic, linoleic acid, and alpha linolenic acid. For resorcinol, low nitrogen decreased palmitoleic, and stearic acid, resulting in higher oleic acid. Phenol was relatively unaffected by low nitrogen conditions.

**Table 3:** Fatty acid composition profile (%) for cells grown in high nitrogen (TOHN) and low nitrogen (TOLN) media containing 1 g/L carbon

Substrate	N conc. (g/L)	C16:0	C16:1	C18:0	C18:1	C18:2	C18:3
Glucose	4	8.9 ± 1.2	14.2 ± 0.4	6.6 ± 0.4	19.7 ± 0.4	41.4 ± 2.1	9.2 ± 0.1
Resorcinol	4	9.1 ± 0.5	14.5 ± 0.6	6.5 ± 0.2	31.6 ± 1.1	38.3 ± 2.1	0.0 ± 0.0
pHBA	4	10.1 ± 0.7	14.2 ± 0.4	7.6 ± 0.7	30.8 ± 1.3	36.0 ± 0.6	1.4 ± 1.9
Phenol	4	8.6 ± 0.5	11.1 ± 0.3	6.1 ± 0.6	37.9 ± 3.5	36.3 ± 2.2	0.0 ± 0.0
Glucose	0.012	12.5 ± 0.4	17.4 ± 0.5	8.0 ± 0.3	26.2 ± 0.3	29.3 ± 0.4	6.7 ± 0.1
Resorcinol	0.012	8.5 ± 4.4	9.9 ± 4.9	0.0 ± 0.0	43.1 ± 6.4	38.5 ± 2.8	0.0 ± 0.0
pHBA	0.012	18.0 ± 0.5	13.4 ± 0.4	4.0 ± 2.9	31.9 ± 1.7	32.6 ± 1.4	0.0 ± 0.0
Phenol	0.012	7.5 ± 0.5	13.1 ± 0.1	6.9 ± 0.2	39.5 ± 1.2	32.9 ± 1.2	0.0 ± 0.0

Errors are reported as standard deviation of biological triplicates

Amongst the three aromatic compounds used in the metabolism studies, resorcinol could be used at a concentration of 3 g/L, the highest concentration with no change in the growth or health of the cells (data not shown). Therefore, we used this substrate in subsequent experiments to improve lipid accumulation from aromatics and to test different feeding strategies to effectively increase the C:N ratio.

### Co-utilization of carbon sources results in diauxic growth

**Fig. 3:**
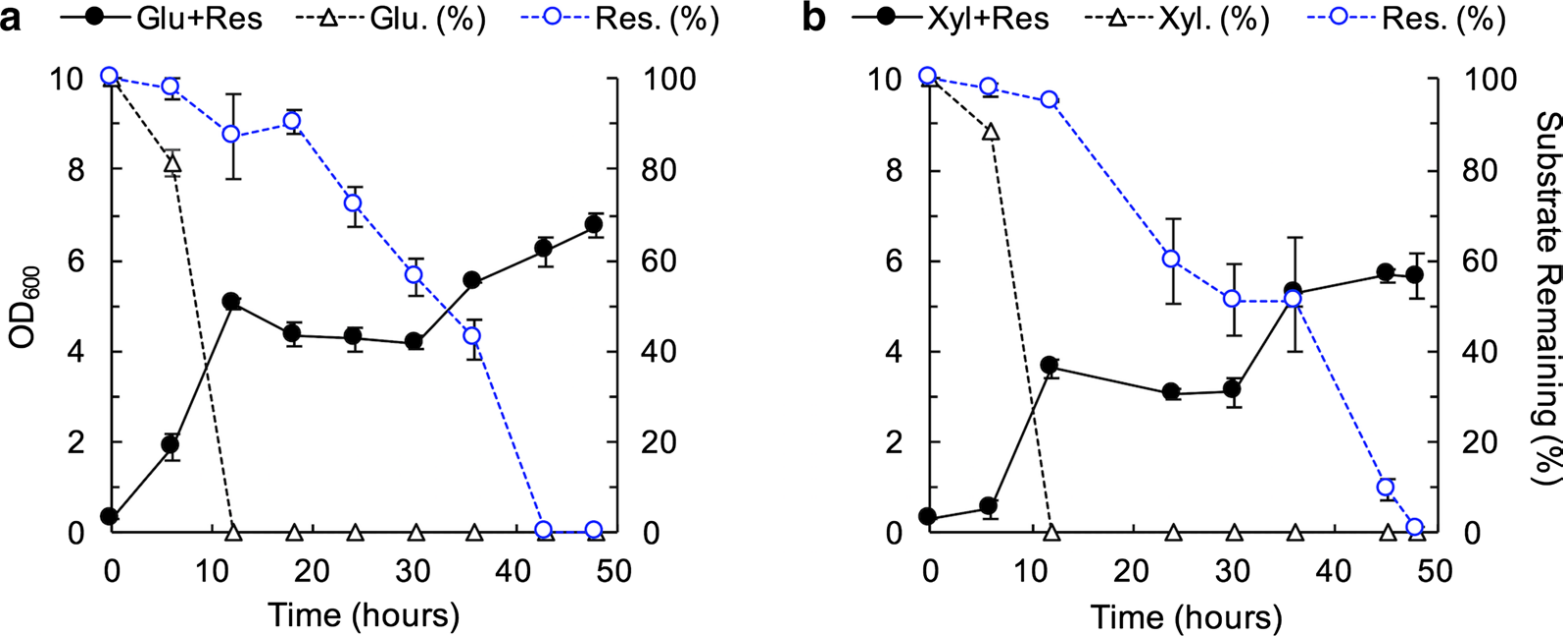
Diauxic growth of *T. oleaginosus* cells when cultured in dual-carbon media. **a** TOLN media containing 1.5 g/L glucose + 1.5 g/L resorcinol and **b** TOLN media containing 1.5 g/L Xylose + 1.5 g/L resorcinol. Growth (closed circles, solid lines) and substrate utilization (open markers, dashed lines) for sugars (black) and resorcinol (blue). The left axis represents OD_600_ while the right axis represents percent substrate remaining in the media. Sugars are preferentially consumed, although resorcinol is partially consumed by hour 12 in both mixed carbon media. The cell density remains unchanged for 18 h after exhaustion, resulting in diauxic growth. The data are the mean and error bars are standard deviation of biological replicates (n = 3). Lines are used only for visual aid

Consolidated bioprocessing involving simultaneous biomass degradation and substrate utilization would contain sugars, such as glucose and xylose, and lignin-derived components; therefore, we measured growth in mixed carbon sources. *Trichosporon oleaginosus* demonstrates diauxic growth when grown in low nitrogen media comprised of 1.5 g/L each of sugar (either glucose or xylose) and resorcinol (Fig. 3). Substrate utilization data show preferential metabolism of the sugars by hour 12, followed by resorcinol; however, the slow consumption of resorcinol during the first 12 h suggests some degree of simultaneous utilization. The consumption rate of resorcinol increases significantly once the sugars are fully consumed. The resulting growth after glucose or xylose is fully consumed has a long lag phase until at least hour 30, when cell growth continues. This observation is in contrast to *T. oleaginosus* growth in media containing both xylose and glucose, where glucose substrate was preferentially consumed, but the growth rates were similar between both carbon sources (Additional file 1: Figure S1). Measurements of cell density and lipid accumulation show no significant difference in percent lipid accumulation between xylose + resorcinol media, xylose media, and resorcinol media; however, lipid accumulation percentage in glucose was lower than resorcinol and both were lower than mixed glucose + resorcinol media (Table 4). However, it should be noted that due to the use of a small amount of yeast extract, the C:N value of 36:1 is still somewhat low, discouraging significant lipid accumulation. Lipid profiles show consistent distribution of fatty acids across various carbon sources (Additional file 1: Table S1).

**Table 4:** Measurement of dry cell weight, lipid titer, and percentage lipid accumulation for cells grown in dual-carbon media and comparison to single carbon media

Substrate	C conc. (g/L)	N conc. (g/L)	Dry cell weight (g/L)	Lipid titer (g/L)	Lipid accumulation (%)
Xylose	3.0	0.0012	1.6 ± 0.0	0.23 ± 0.0	14.5 ± 0.7
Glucose	3.0	0.0012	1.2 ± 0.0	0.10 ± 0.0	8.2 ± 0.2
Resorcinol	3.0	0.0012	1.0 ± 0.1	0.11 ± 0.0	11.3 ± 1.0
Xyl + Res	1.5/1.5	0.0012	1.2 ± 0.4	0.14 ± 0.0	12.6 ± 3.1
Glu + Res	1.5/1.5	0.0012	0.7 ± 0.0	0.10 ± 0.0	13.9 ± 1.7

Errors are reported as standard deviation of biological triplicates

### *T. oleaginosus* remains oleaginous while metabolizing high concentration of aromatics

**Fig. 4:**
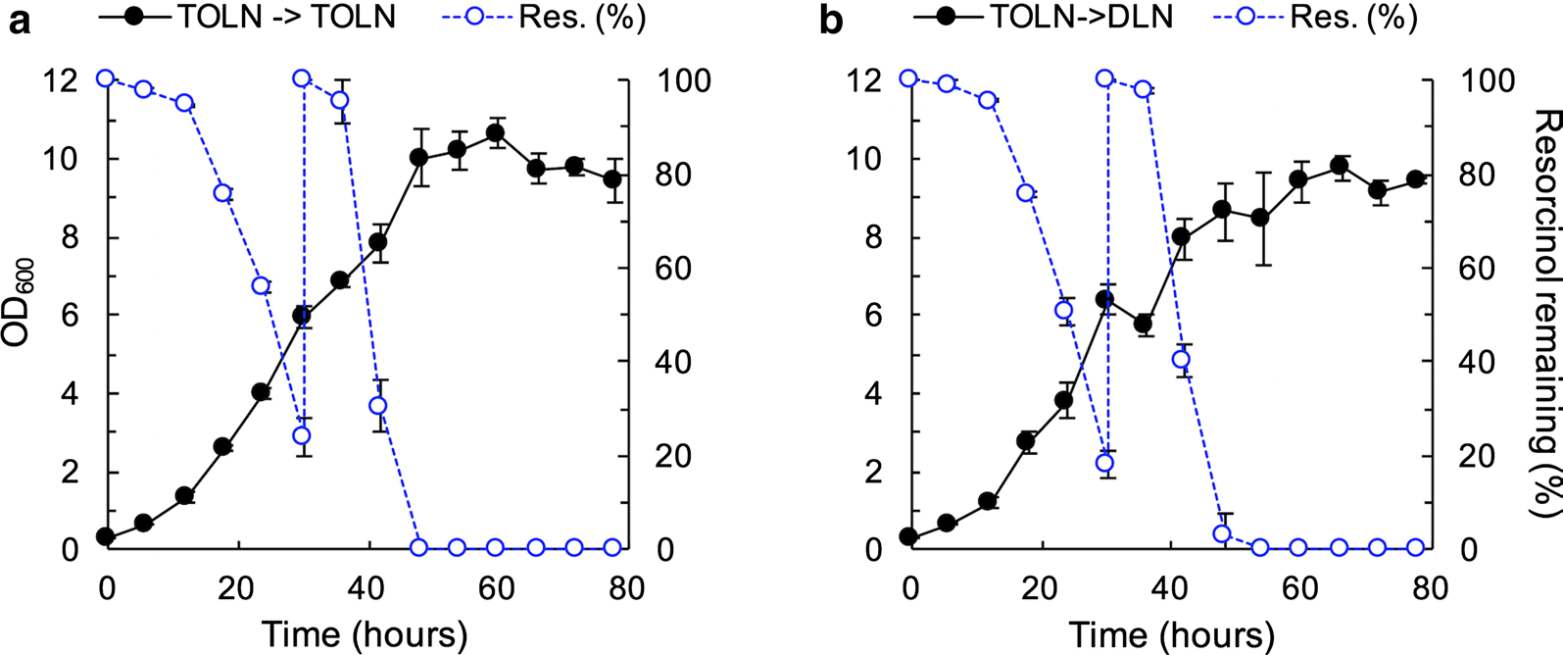
Two-stage feeding cultures demonstrate complete consumption of resorcinol and no metabolic limitations. Growth (closed circles, solid lines) is on the left axis and substrate utilization (open circles, dashed lines) is on the right axis. **a** Cells are grown for 30 h in TOLN media containing 3 g/L resorcinol (C:N ratio = 36:1). After 30 h, cells are switched into fresh TOLN media containing 3 g/L resorcinol (C:N = 36:1) and grown for an additional 48 h. **b** Cells are grown for 30 h in TOLN media containing 3 g/L resorcinol (C:N ratio = 36:1). After 30 h, cells are switched into fresh defined low nitrogen (DLN) media containing 3 g/L resorcinol (C:N = 361:1) and grown for an additional 48 h. The data are the mean and error bars are standard deviation of biological replicates (n = 3). Lines are used only for visual aid

As noted earlier, 3 g/L was the highest concentration of resorcinol tested that did not result in a lower growth rate. While non-inhibited growth on this concentration of resorcinol is already high compared to other yeast [37], we sought to determine if more substrate could be utilized using alternative feeding strategies. The first attempt feed more resorcinol was through a two-stage feeding strategy where *T. oleaginosus* cells were grown for 30 h in TOLN containing 3 g/L resorcinol to accumulate biomass. The time of 30 h was chosen because the cells are in late exponential phase at this point (Fig. 4). Cells were harvested and resuspended in fresh TOLN (C:N = 36:1) or with fresh defined low nitrogen (DLN) media (C:N = 361:1), both containing 3 g/L resorcinol and grown for another 48 h. DLN media use was possible once cell biomass was obtained in the first stage, and the yeast extract could be omitted from the TOLN media. The DLN C:N ratio was altered to match that of previously established C:N ratios [40] by eliminating yeast extract and supplementing nitrogen in the form of ammonium sulfate. Two-stage feeding is required because DLN alone does not promote significant biomass accumulation (0.12 ± 0.0 g/L cell density after 80 h). The higher C:N ratio was better for lipid accumulation (Table 5). Switching into a lipid accumulating media results in a 60% improvement in lipid accumulation from 14.5 ± 0.9 to 35.9 ± 1.2% (Table 5) while maintaining a similar lipid composition profile similar to that of previous experiments (Additional file 1: Table S2).

**Table 5:** Measurement of dry cell weight, lipid titer, and percentage lipid accumulation for two-stage and fed-batch cultures

Sample	Final C conc. (g/L)	Final N conc. (g/L)	Dry cell weight (g/L)	Lipid titer (g/L)	Lipid accumulation (%)
TOLN → TOLN	6.0	0.00120	1.81 ± 0.0	0.26 ± 0.1	14.5 ± 0.9
TOLN → DLN	6.0	0.00012	1.48 ± 0.1	0.53 ± 0.0	35.9 ± 1.2
Fed batch	11.0	0.00120	2.36 ± 0.2	1.64 ± 0.2	69.5 ± 4.0

Errors are reported as standard deviation of biological triplicates

### *T. oleaginosus* accumulates greater than 69% of its biomass as lipids in a fed-batch shake flask using resorcinol as a sole carbon source

**Fig. 5:**
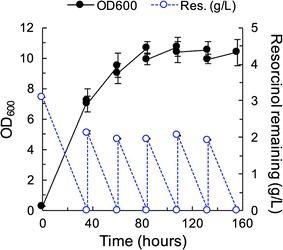
Fed-batch feeding strategy results in significant resorcinol metabolism over a prolonged period. Growth (closed circles, solid lines) are on the left axis and substrate utilization (open circles, dashed lines) are on the right axis. Initial TOLN media contained 3 g/L resorcinol. After 36 h, cells were fed with 2 g/L resorcinol in 24 h intervals while keeping culture volume constant. Cultures show full resorcinol consumption after each feeding; however, cell growth plateaus, potentially indicating high lipid accumulation in the late phase of culturing. The data are the mean and error bars are standard deviation of biological replicates (n = 5). Lines are used only for visual aid

Cells for the fed-batch experiment were initially inoculated to an OD of 0.3 in TOLN containing 3 g/L of resorcinol and were grown for 36 h. After this time, 2 g/L of resorcinol were fed every 24 h while keeping the culture volume constant (Fig. 5). In this manner, nutrients such as nitrogen were depleted over time, and only carbon was replenished with every feeding. A total of 11 g/L of resorcinol was delivered over 160 h, as compared to 3 g/L over 48 h in batch experiments, resulting in a 73% improvement in total carbon fed. Fed-batch feeding results in 87.4% improvement in total lipid titer from 0.11 ± 0.0 g/L (Table 4) to 1.64 ± 0.2 g/L (Table 5). Lipid accumulation increased from 11.3 ± 0.0% (Table 4) to 69.5 ± 4.0% (Table 5), resulting in an 87.4% improvement. When compared to the two-stage feeding described earlier, lipid titer improved by 39.1% and lipid accumulation improved by 38.8%. The lipid profile was similar to those found in two-stage feeding experiments (Additional file 1: Table S2).

## Discussion

*Trichosporon oleaginosus* has been studied for its ability to accumulate a significant amount of lipids from a variety of feedstocks. This is the first report showing that *T. oleaginosus* is able to metabolize model lignin monoaromatic compounds when used as a sole carbon source. It is likewise the first report of *T. oleaginosus* metabolizing aromatics in mixed sugar and aromatic substrates. While other yeast have been shown to tolerate and metabolize aromatic compounds [41], this is the first demonstration of yeast growing on aromatics and accumulating lipids greater than 20% on a dry weight basis. In fed-batch experiments, *T. oleaginosus* was able to accumulate over 69% of its dry cell weight as lipids using aromatic compound resorcinol as the sole carbon source, demonstrating its promise as a model organism for aromatic to oleochemical conversion.

To date, the highest reported lipid accumulation from *T. oleaginosus* is 73.4% on a dry weight basis using acetate as the sole carbon source [42]. This lipid accumulation compares favorably to the lipid accumulation of 69.5% on a dry weight basis from resorcinol demonstrated in this work. We were unable to find any report of lipid accumulation in a microbe grown on resorcinol as the sole carbon source. The literature has focused on bioremediation of xenobiotics and anthropomorphic aromatic substrates rather than lipid accumulation. Amongst oleaginous bacteria, *Rhodococcus opacus* strains DSM 1069 and PD630, accumulate up to 20% of dry weight as lipids when grown on pHBA and vanillic acid in optimized fed-batch reactors [8]. Phenol toxicity impacted the growth rate when reaching 0.3 g/L [9]. A strain of *R. opacus* PD630 evolved for higher tolerance, increasing from 0.3 g/L up to 1.5 g/L achieving lipid accumulation of 11.7% on a dry weight basis [9]. The titer was not reported, but could not be higher than 1.5 g/L, the concentration of phenol used in the experiments, and theoretically must be much lower. By comparison, our highest lipid titer was 1.64 ± 0.2 g/L with a lipid accumulation of 69.3% and was achieved using a wild-type strain. *Trichosporon oleaginosus* is able to accumulate significantly higher titers of lipids when grown on higher concentrations of preferred substrates such as glucose [38, 39, 43]; however, the toxicity of aromatic compounds limits the concentration of aromatics dosed at a single time. The toxicity limitation of aromatic substrates limits the carbon to nitrogen (C:N) ratio when growing initial cell biomass, which benefits significantly from a small amount of yeast extract. In one study, *T. oleaginosus* was previously shown to achieve the highest percent accumulation and lipid titer at a C:N ratio of 99 [44], whereas another study used a C:N ratio of 360 [40]. These high C:N ratios are consistent with our observations of poor lipid accumulation using a C:N ratio of 36, but drastically greater lipid accumulation using a ratio of 360:1 in fed-batch experiments, emphasizing the role of the C:N ratio on lipid accumulation in *T. oleaginosus*.

In nature, lignin can be enzymatically depolymerized to a wide variety of aromatics. Similarly, catalytic depolymerization also results in a heterogeneous mixture of aromatic compounds [41]. Funneling pathways overcome the inherent heterogeneity of lignin by converting key aromatic compounds to important intermediates for the TCA cycle [4, 20–23]. The beta-ketoadipate pathway is well-conserved amongst aromatic metabolizing bacteria and yeast. The most common pathways are intra-diol (ortho) ring cleavage and extra-diol (meta) ring cleavage pathways. Based on our demonstration of resorcinol, pHBA, and phenol metabolism, *T. oleaginosus* appears to have each of the three major funneling pathways. The enzymatic activity of aromatic metabolism was previously studied in *Trichosporon cutaneum.* Metabolite analysis demonstrated ortho ring cleavage reaction products and the absence of meta ring cleavage products [45, 46]. Given both are from the same genus, it is likely that *T. oleaginosus* uses the ortho ring cleavage pathway. A more detailed study is needed to definitively identify the pathways for aromatic metabolism.

Lignocellulosic hydrolysates contain sugars derived from the cellulose and hemicellulose content and aromatics from the lignin content; therefore, our demonstration that *T. oleaginosus* co-utilizes resorcinol and sugars (glucose or xylose) establishes the potential to utilize multiple components of lignocellulosic biomass. Given that *T. oleaginosus* can co-utilize glucose and xylose (Additional file 1: Figure S1), we expect that this yeast will co-utilize glucose, xylose, and aromatics. In addition, this finding suggests it may be unnecessary to remove phenolic content in recalcitrant feedstocks, as is typically required due aromatic toxicity and lack of metabolism [47]. Related organisms show similar co-utilization. In fact, wild type *T. cutaneum* was shown to simultaneously uptake glucose and phenol, with the rate of glucose utilization being much higher than that of phenol utilization [48], consistent with our findings. The presence of glucose decreased phenol utilization and catechol-1,2 dioxygenase (an enzyme belonging to the catechol funneling pathway) activity by 60 and 75%, respectively.

We show *T. oleaginosus* is able to tolerate 15 different compounds to various degrees, with MICs as high as 15.5 g/L for protocatechuate and as low as 0.2 g/L for vanillin. Improving tolerance will be important for further development of industrially relevant strains. Several mechanisms have been reported to avoid aromatic toxicity including dynamic lipid distribution, efficient transporters, and highly active aromatic conversion genes. The mechanism for aromatic tolerance in bacteria is mostly attributed to redistribution of the highly dynamic lipid composition and the activation of aromatic efflux transporters [49–53]. *Pseudomonas putida* strains isolated from toluene-polluted sites responded to aromatic-induced membrane stress by increasing the ratio of *trans* and *cis* fatty acid isomers, making the cell membrane more rigid. *Pseudomonas putida* DOT-T1E has been shown to tolerate and metabolize 17 g/L pHBA, which the authors attribute to the cell membrane rigidity [54]. A more recent study showed the fatty acid composition of *R. opacus* strains grown on phenol and benzene also exhibited increased *trans*-fatty acids and increased 10-methyl branched fatty acid content in the presence of aromatics [51]. While these specific membrane adaptations are unique to prokaryotes, eukaryotes can likewise alter membrane fluidity through alterations in composition and sterols [55]. Aside from altering membrane fluidity, strains of *P. putida* have been shown to utilize efflux pumps to remove toluene from the cell membrane [50, 53]. However, efflux pumps would be counter to the desired aromatic metabolism. Recently, an evolved strain of *R. opacus* PD630 showed increased importer activity led to faster growth on phenol, and higher tolerance to phenol compared to the wild type strain [9]. Lastly, a recent study in *Saccharomyces cerevisiae* shows that enabling efficient conversion genes also improves tolerance by removing the toxic compounds and converting them to a more benign intermediate [56].

We hypothesize that *T. oleaginosus* uses a combination of these mechanisms. The fatty acid distribution changes in the presence of aromatic compounds as compared to glucose, with glucose resulting in C18:3, but aromatic grown cells have a higher content of C18:1 and C18:2. The identical growth rates of cells using glucose, pHBA, or resorcinol suggest the potential for efficient metabolism of aromatics. Enhancing import or metabolism of aromatics could lead to improved tolerance. The recent demonstration of genetic engineering tools for *T.* *oleaginosus* [38] makes it possible to engineer increased tolerance through control of membrane fluidity and overexpression of rate limiting aromatic metabolism genes. Improving aromatic tolerance in this, and any microbe, is necessary to economically utilize higher concentrations of substrate for industrial applications, further motivating future studies understanding the mechanism for aromatic metabolism and tolerance and engineering improvements.

## Conclusions

*Trichosporon oleaginosus* is the first yeast shown to metabolize aromatics and remain oleaginous, accumulating nearly 70% of its biomass as lipids when grown on resorcinol in fed-batch. This yeast shows promise for utilizing aromatic-containing feedstocks, such as lignin and wastewater effluent, for microbial production of oleochemicals or aromatic-derived compounds due to its natural ability to tolerate and metabolize relatively high concentrations of aromatics. A complete understanding coupled to additional metabolic engineering tools will enable pathway engineering for improving tolerance to and conversion of aromatics for industrial applications. Nevertheless, this yeast is well-positioned to become a model system for aromatics metabolism to lipids and oleochemicals.

## Methods and materials

### Reagents

A list of all chemical reagents used and the source of these chemicals is listed in Additional file 1: Table S3. All enzyme reagents were purchased from New England Biolabs (Ipswich, MA) unless otherwise stated. *Trichosporon oleaginosus* was obtained from the American Type Culture Center (ATCC^®^ 20509™).

### Tolerance studies

Tolerance studies were performed in 48-well plates (Nunclon^®^ 48-well plate) in a Biotek^®^ Synergy™ Mx multimode microplate reader. 2 mL YPD pre-cultures were grown overnight and used to inoculate 250 μL YPD (10 g/L yeast extract, 20 g/L peptone, 20 g/L glucose) containing various aromatic compounds described in Table 1 to an OD = 0.3. Cells were grown over 48 h in a 48 well plate with the lid on, fast shake speed, and at 28 °C. The plate reader scanned every hour at 600 nm. Studies were performed at least in triplicate. Hydroxyquinol and catechol resulted in media color changes that prevented use of the spectrophotometric measurement, so these samples were plated to assess CFUs.

### Aromatic growth studies

Single and dual carbon source cultures were cultured in the same fashion. *Trichosporon oleaginosus* (ATCC^®^ 20509™) cells were grown in 2 mL YPD pre-cultures overnight. Cells were washed three times with new media, and inoculated to an initial OD_600_ of 0.3 in 50 mL baffled Erlenmeyer Corning^®^ flasks containing 15 mL of either high nitrogen (TOHN) or low nitrogen (TOLN) media (modified from [40]) with appropriate carbon source and concentration. Details on media composition are included in Additional file 1: Table S4. Cell washing entailed centrifuging cells at 1100×*g* for 4 min at 4 °C, decanting supernatant, and re-suspending in destination media. Carbon sources included glucose, xylose, phenol, resorcinol, and 4-hydroxybenzoic acid (pHBA). Optical density (OD) readings were measured on a Thermo Scientific NanoDrop™ 2000 at 600 nm and corrected by a factor of 17.725. All experiments were performed in triplicate.

Two-stage feeding and fed-batch cultures were started in the same manner. After biomass accumulation in the two-stage feeding experiments, the whole cell culture was transferred to a 50 mL centrifuge tube, spun down at 4000 rpm for 3 min at 4 °C, and re-suspended in 15 mL fresh TOLN or DLN (Additional file 1: Table S5) media. The entire 15 mL was transferred back to the 50 mL baffled flask and cultured for the 48 h lipid accumulation phase. For fed-batch experiments, 1.5 mL of cell culture was removed after the initial biomass accumulating phase. Stock resorcinol (20 g/L in ddH_2_O) was added to feed 2 g/L at a time. This removal of biomass and addition of stock resorcinol was repeated every 24 h until the end of the experiment. All experiments were performed in triplicate.

### Dry cell weight and lipid analysis

Cells were harvested for dry cell weight by washing 10 mL of cell culture with 20 mL of ddH_2_O three times and drying overnight at 40 °C under vacuum in aluminum pans. Dry cell weights were measured using an analytical balance. To identify and quantify lipids in cell biomass, extracted cellular lipids were transesterified to FAMEs as described previously with minor modifications. Briefly, 1 mL cell culture was harvested and spun down at 13,000 rpm for 3 min at 25 °C. 100 μL glyceryl triheptadecanoate at a concentration of 2 mg/mL methanol was added to the cell pellet as an internal standard. Lipids were transesterified to FAMEs with 500 μL of 0.5 N sodium methoxide followed by 30 min of vortexing at 2000 rpm. The solution was neutralized with 40 μL sulfuric acid. FAMEs were extracted by adding 850 μL hexane followed by 20 min of vortexing at 2000 rpm. The mixture was centrifuged for 1 min at 8000 rpm, and 800 μL of the organic layer was collected for GC-FID analysis (Agilent 7890B) and quantification [32].

### Substrate utilization

Aromatic substrate utilization was analyzed using a Waters 600E multisolvent delivery (Waters Corporation) high performance liquid chromatography (HPLC) system with a BioRad Fast Acid Analysis HPLC column with 10% v/v acetonitrile and 0.01 N H_2_SO_4_ in a 1:1 mixture as the eluent, a flow rate 0.6 mL/min, temperature of 65 °C, and with a Waters 996 PDA detector. Phenol was detected at 270 nm, resorcinol at 274 nm, and pHBA at 254 nm. Glucose and xylose were measured using the same column at 85 °C, 5 mM H_2_SO_4_ eluent, and a Waters 2414 refractometer. Concentrations were calculated from standard curves created for each carbon source in the appropriate medium.

## Additional file

**Additional file 1.** Additional data related to dual-carbon source experiments and methods. **Figure S1.** Growth curves and substrate consumption data for glucose, xylose, resorcinol, and glucose/xylose. **Table S1.** Dual-carbon source fatty acid distribution. **Table S2.** Two-stage and fed-batch fatty acid composition. **Table S3.** List of chemical suppliers. **Tables S4**, **S5.** Media composition.

## Abbreviations

TOHN: *Trichosporon oleaginosus* high nitrogen
TOLN: *Trichosporon oleaginosus* low nitrogen
DLN: defined low nitrogen
pHBA: 4-hydroxybenzoic acid
HPLC: high performance liquid chromatography
GC-FID: gas chromatography-flame ionization detection
