# Supplementary material for: Metabolism of aromatics by Trichosporon oleaginosus while remaining oleaginous
Source: Microb Cell Fact. 2017 Nov 17;16:206. doi: 10.1186/s12934-017-0820-8 (PMC5693591; doi:10.1186/s12934-017-0820-8)
Supplement: Supplementary file 1 — Additional file 1. Additional data related to dual-carbon source experiments and methods. Figure S1. Growth curves and substrate consumption data for glucose, xylose, resorcinol, and glucose/xylose. Table S1. Dual-carbon source fatty acid distribution. Table S2. Two-stage and fed-batch fatty acid composition. Table S3. List of chemical suppliers. Tables S4, S5. Media composition. [file 12934_2017_820_MOESM1_ESM.docx]

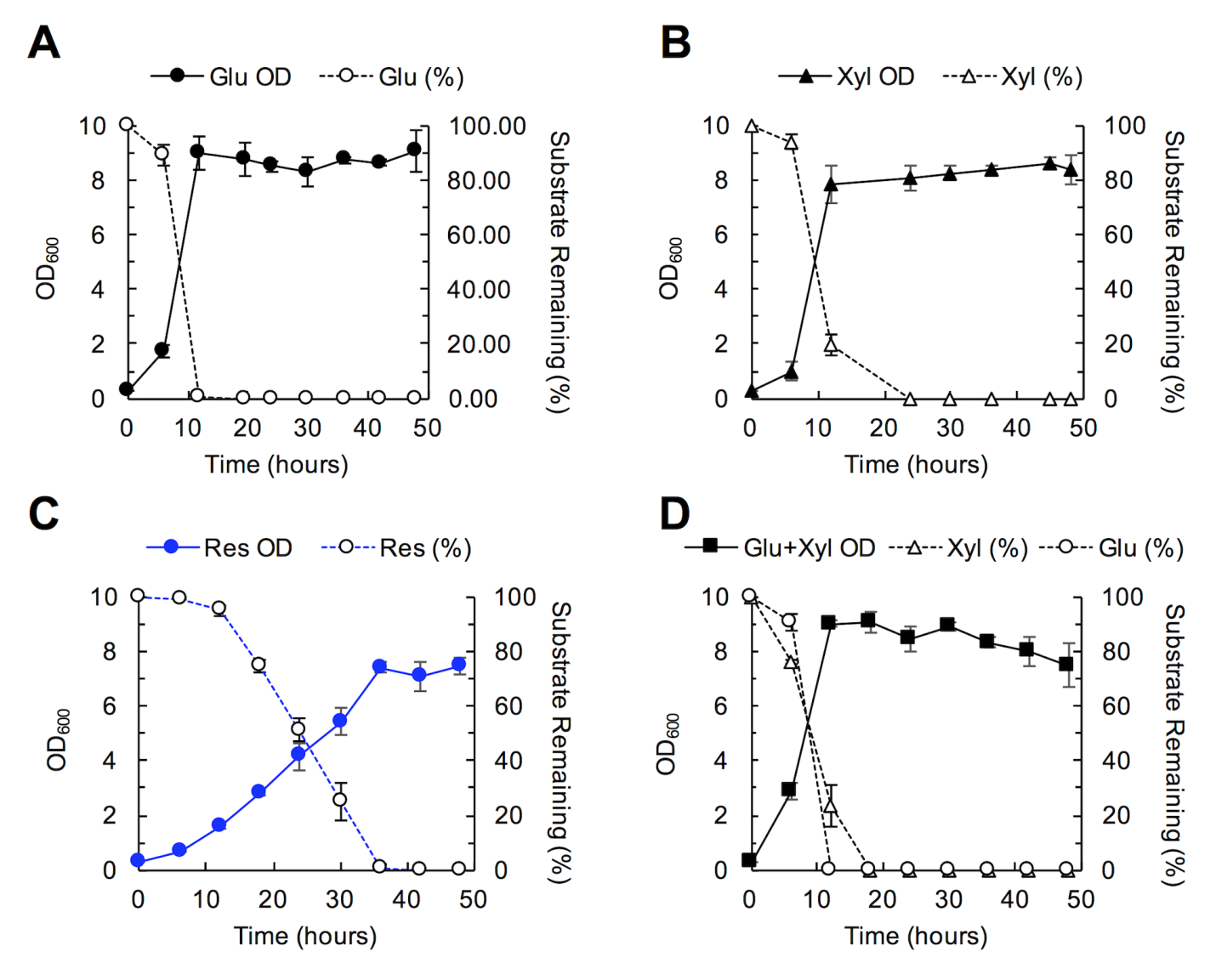
Figure S1. Growth (closed marker, solid lines) and substrate utilization (open marker, dashed lines) data for A) glucose, B) xylose, C) resorcinol, and D) glucose + xylose in TOLN media. All carbon is at a total concentration of 3 g/L. The left axis represents OD_600_ while the right axis represents percent substrate remaining in the media. Each panel shows full consumption of the compounds. The data in panels A-D are the mean and error bars are standard deviation of biological triplicates. Lines are used only for visual aid.

Table S1. Fatty acid composition (%) for cells grown in dual-carbon source media.

| **Substrate** | **C Conc. (g/L)** | **C16:0** | **C16:1** | **C18:0** | **C18:1** | **C18:2** | **C18:3** |
| --- | --- | --- | --- | --- | --- | --- | --- |
| Xylose | 3.0 | 1.9 ± 0.2 | 21.6 ± 0.1 | 16.9 ± 0.2 | 41.8 ± 0.6 | 15.7 ± 0.1 | 3.0 ± 0.0 |
| Glucose | 3.0 | 3.2 ± 0.6 | 21.2 ± 0.8 | 14.5 ± 0.8 | 41.8 ± 1.2 | 15.7 ± 0.6 | 2.7 ± 0.1 |
| Resorcinol | 3.0 | 2.5 ± 0.4 | 19.5 ± 0.3 | 17.6 ± 1.0 | 47.5 ± 1.0 | 13.0 ± 1.0 | 0.0 ± 0.0 |
| Xyl + Res | 1.5 / 1.5 | 3.0 ± 0.3 | 17.1 ± 0.7 | 20.5 ± 0.5 | 46.5 ± 0.8 | 12.0 ± 0.6 | 0.9 ± 0.6 |
| Glu + Res | 1.5 / 1.5 | 3.4 ± 0.3 | 17.6 ± 0.6 | 18.5 ± 0.4 | 46.4 ± 1.3 | 12.8 ± 0.2 | 1.4 ± 0.1 |

Errors are reported as standard deviation of biological triplicates.

Table S2. Fatty acid composition (%) for two-stage and fed-batch cultures.

| **Sample** | **Final C conc. (g/L)** | **C16:0** | **C16:1** | **C18:0** | **C18:1** | **C18:2** | **C18:3** |
| --- | --- | --- | --- | --- | --- | --- | --- |
| TOLN->TOLN | 6.0 | 2.1 ± 0.1 | 19.9 ± 0.3 | 13.1 ± 0.3 | 46.7 ± 0.4 | 17.1 ± 0.3 | 1.0 ± 0.1 |
| TOLN->DLN | 6.0 | 1.0 ± 0.1 | 23.7 ± 0.1 | 15.3 ± 0.3 | 51.5 ± 0.6 | 8.1 ± 0.4 | 0.5 ± 0.0 |
| Fed batch | 11.0 | 0.3 ± 0.0 | 20.3 ± 1.1 | 23.3 ± 1.0 | 50.2 ± 1.2 | 5.7 ± 0.4 | 0.2 ± 0.0 |

Errors are reported as standard deviation of biological triplicates.

Table S3. List of chemical suppliers

| **Chemical** | **Supplier** |
| --- | --- |
| 4-hydroxybenzoic acid | Alfa Aesar |
| 2,4-dihydroxybenzoic acid | Acros Organics |
| Ammonium Sulfate | Fisher Scientific |
| Benzaldehyde | Alfa Aesar |
| Benzoic acid | Sigma Aldrich |
| Calcium chloride hexahydrate | Sigma Aldrich |
| Catechol | Alfa Aesar |
| Copper (II) sulfate pentahydrate | VWR |
| Ferulic acid | MP Biomedicals |
| Glucose | Sigma Aldrich |
| Guaiacol | Spectrum |
| Hydroxyquinol | Fisher Scientific |
| Magnesium sulfate heptahydrate | Amresco, Inc. |
| Manganese (II) chloride tetrahydrate | Alfa Aesar |
| p-coumaric acid | Sigma Aldrich |
| p-cresol | TCI America |
| Phenol | Amresco, Inc. |
| Potassium phosphate dibasic | Amresco, Inc. |
| Protocatechuate | Alfa Aesar |
| Resorcinol | Acros Organics |
| Syringic acid | Acros Organics |
| Vanillin | EM science |
| Vanillic acid | Alfa Aesar |
| Yeast Extract | BD Bacto |
| Zinc (II) sulfate heptahydrate | Amresco, Inc. |

Table S4. Media composition for high nitrogen (TOHN) and low nitrogen (TOLN) conditions.

|  | TOHN | TOLN | Units |
| --- | --- | --- | --- |
| Carbon Source | X | X | g/L |
| Yeast Extract | 0.75 | 0.75 | g/L |
| (NH_4_)_2_SO_4_ | 4 | 0.0012 | g/L |
| MgSO_4_ • 7H_2_0 | 1.5 | 1.5 | g/L |
| KH_2_PO_4_ | 0.4 | 0.4 | g/L |
| CaCl_2_ • 2H_2_O | 0.22 | 0.22 | g/L |
| ZnSO_4_ • 7H_2_O | 0.55 | 0.55 | μg/L |
| MnCl_2_ • 4H_2_O | 24.2 | 24.2 | μg/L |
| CuSO_4_ • 5H_2_O | 25 | 25 | μg/L |

Table S5. Media composition for defined low nitrogen (DLN) conditions.

|  | DLN | Units |
| --- | --- | --- |
| Carbon Source | 3.0 | g/L |
| (NH_4_)_2_SO_4_ | 1.66 | mg/L |
| MgSO_4_ • 7H_2_0 | 1.5 | g/L |
| KH_2_PO_4_ | 0.4 | g/L |
| CaCl_2_ • 2H_2_O | 0.22 | g/L |
| ZnSO_4_ • 7H_2_O | 0.55 | ug/L |
| MnCl_2_ • 4H_2_O | 24.2 | ug/L |
| CuSO_4_ • 5H_2_O | 25.0 | ug/L |
